# Splenic artery aneurysm, case series of seven patients

**DOI:** 10.1093/jscr/rjab046

**Published:** 2021-03-25

**Authors:** Javad Salimi, Zahra Omrani, Roozbeh Cheraghali

**Affiliations:** Vascular & Endovascular Surgery, Liver Transplantation Program, Tehran University of Medical Sciences, Tehran, Iran; Iran University of Medical Sciences, Department of Surgery, Rasool Hospital, Tehran, Iran; Vascular & Endovascular Surgery, Liver Transplantation Program, Tehran University of Medical Sciences, Tehran, Iran; Vascular & Endovascular Surgery, Golestan University of Medical Sciences (GOUMS), Gorgan, Iran

## Abstract

Splenic artery aneurysms (SAA) account for 46–60% of all visceral artery aneurysms. Small SAAs are usually asymptomatic, but giant aneurysms are more likely to cause symptoms and can result in life-threatening complications. Treatment of a splenic artery aneurysms includes laparotomy, laparoscopy or endovascular techniques. Case presentation: In this article, seven interesting cases of splenic artery aneurysms in different size and parts of artery and various interventions (open, endovascular and hybrid surgery) are discussed. Six of the patients were male. Five of them had giant SAAs (≥5 cm). Two patients underwent hybrid surgery. Coil embolization was carried out for one patient. All seven patients discharged with no procedure-related complications. Endovascular procedures considered as a first choice of treatment for splenic artery aneurysm. Open surgery is reserved mostly for the treatment of complications or if the endovascular techniques fail, lack of availability of endovascular procedures or allergy to contrast medium.

## INTRODUCTION

The splenic artery is defined as aneurysm when a focal dilatation is observed and its diameter is >50% of the normal vessel diameter [1]. Splenic artery aneurysms (SAAs) account for 46%–60% of all visceral artery aneurysms. Most occur in the distal third of the splenic artery (75%) followed by the middle third (20%). Aneurysms in the proximal splenic artery are uncommon. Small SAAs (≤ cm) are usually asymptomatic, but giant SAAs (≥5 cm) are more likely to cause symptoms and can result in life-threatening complications [2]. Treatment of a SAA includes laparotomy, laparoscopy or endovascular techniques. In recent years, open aneurysm repair of SAAs has been largely replaced by minimally invasive surgery, such as endovascular procedures, which result in less surgical trauma and faster postoperative recovery. However, only selected aneurysms are suitable for these procedures, as marked tortuosity of the artery or SAA in the proximal splenic artery may not be suitable for endovascular management [3–4].

Here we will present and discuss different managements of seven patients with SAAs referred to our institution during 2015–2020.

## CASE PRESENTATION

Six of the patients were male. Five of them had giant SAA (≥5 cm). Two patients underwent hybrid surgery. Coil embolization was done for one patient. All the patients gave their informed consent. Characteristics of patients are shown in Table 1.

**Table 1:** Characteristics of seven patients with SAA

Case	Sex	Age	Size	Intervention	Site of aneurysm	Outcome
1	Female	60	5 cm	Aneurysm resection + splenectomy	Distal	Discharged with no major problem
2	Male	55	6 cm	Aneurysm resection	Middle	
3	Male	52	3 cm	Coil embolization	Middle	Discharged
4	Male	56	6 cm	Hybrid surgery (proximal control by balloon + aneurysm resection)	Proximal	Discharged
5	Male	52	6 cm	Hybrid surgery (proximal control by balloon + aneurysm resection (Fig. 4)	Proximal	Discharged
6	Male	62	4 cm	Aneurysm Resection+ primary arterial anastomosis	Middle	Discharged
7	Male	48	5 cm	Aneurysm resection	Middle	Discharged
						

**Figure 1:**
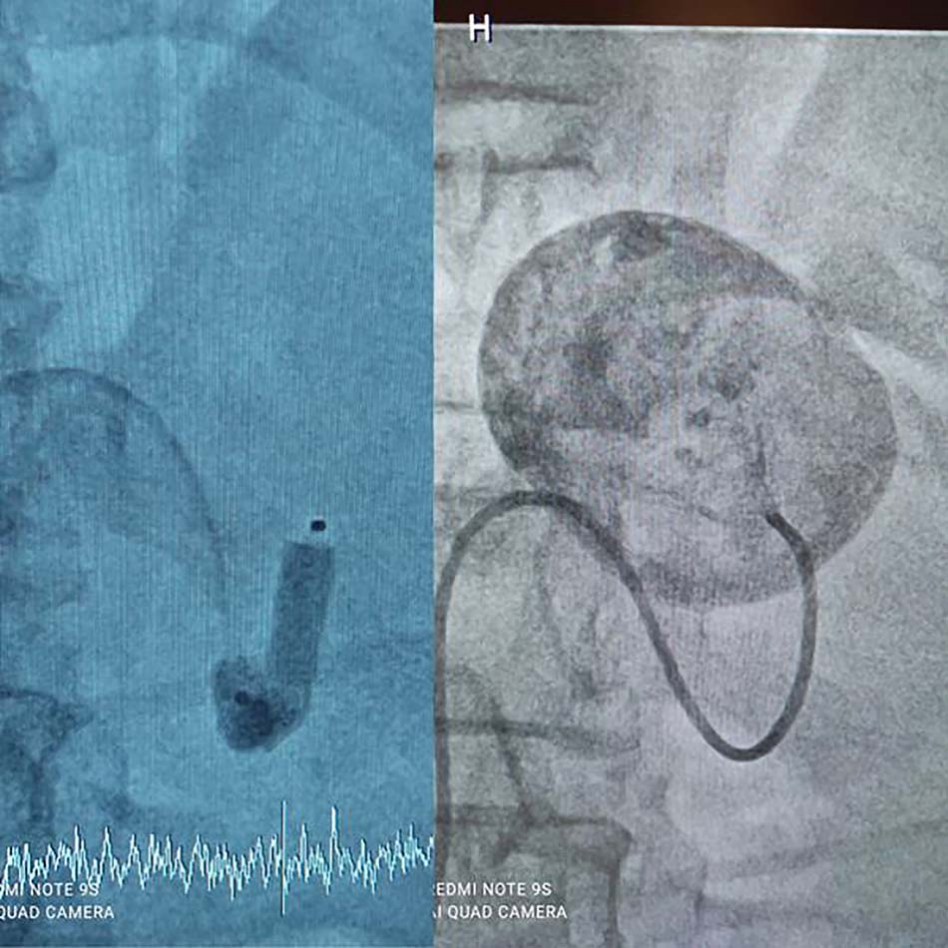
Balloon Proximal control.

**Figure 2:**
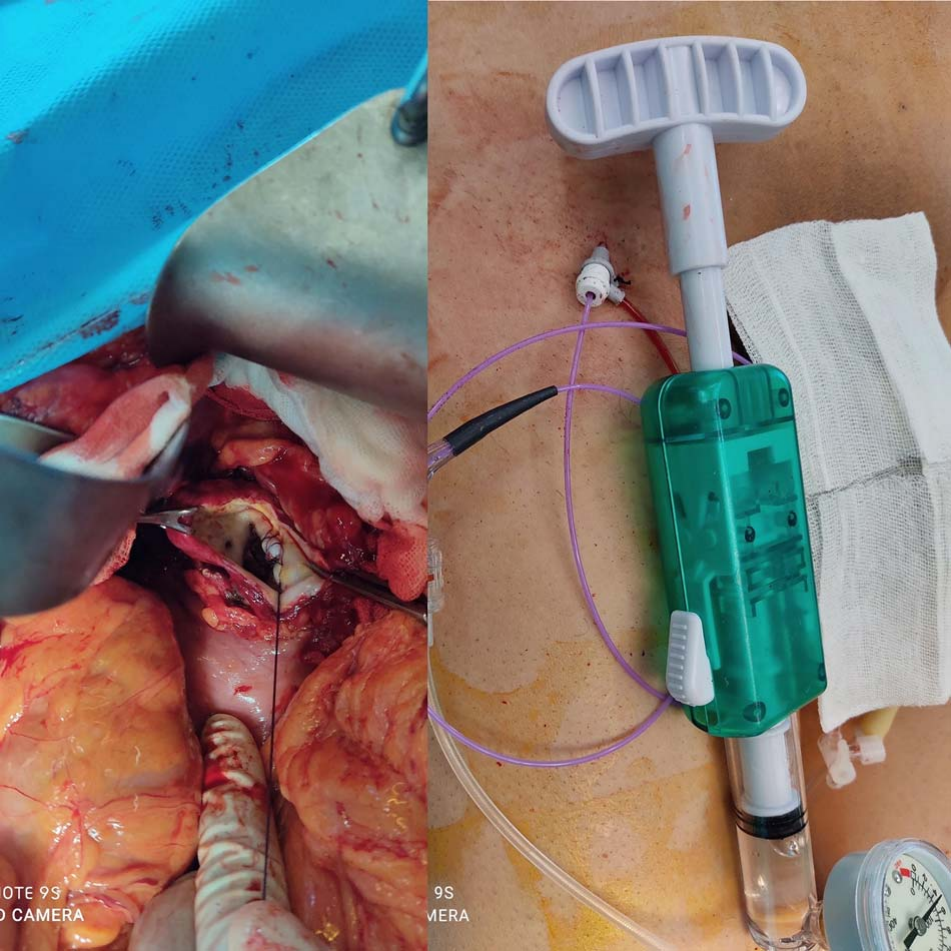
Hybrid surgery: Aneurysm sac (Left), Angiography sheath and inflator (Right).

A 56-year-old male (fourth patient) evaluated for abdominal pain was referred to our hospital. In Computed tomography angiography (CTA) giant proximal SAA was diagnosed. We considered endovascular treatment. Angiography was carried out but because of arterial tortuosity wire did not pass through to the distal part of the artery. Hybrid surgery was the next plan. Balloon (7–40) expanded in proximal part of the aneurysm then in laparotomy, distal part was ligated and proximal part according to the inflated balloon was found easily (Fig. 1). After balloon was deflated, aneurysm was ligated and resected (Fig. 2).

**Figure 3:**
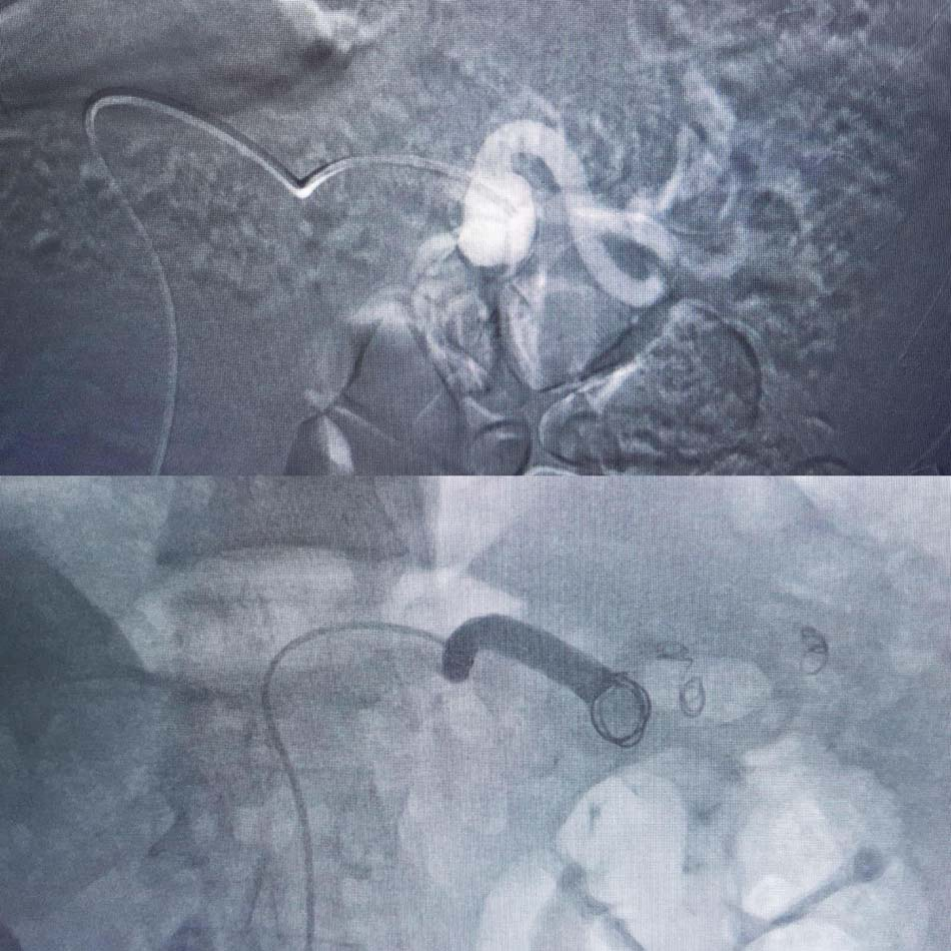
Coil embolization.

In a 52-year-old man (third patient), 3-cm SAA was found incidentally in ultrasound and CTA. According to size of the aneurysm, he was candidate for endovascular treatment. Angiography and positioning of the coils on either side of the aneurysm (‘sandwich technique’) was completed (Fig. 3). Post-embolization checks were performed with selective splenic, celiac and superior mesenteric artery angiograms to confirm occlusion of the main splenic artery and patency of the collateral arteries. All seven patients discharged with no procedure-related complications.

**Figure 4:**
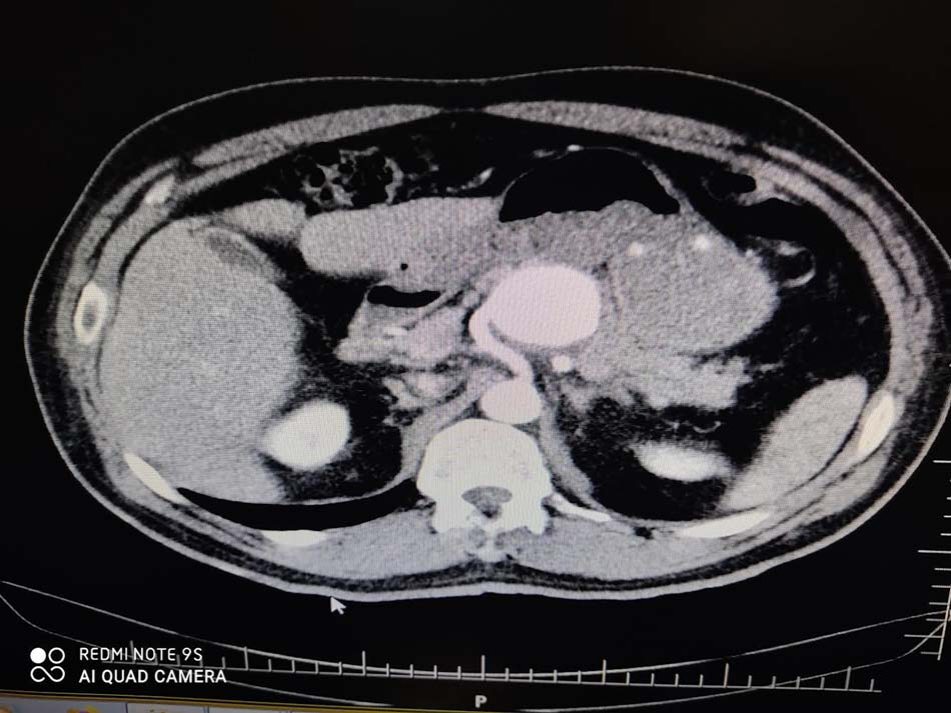
CTA of proximal splenic aneurysm.

## DISCUSSION

Splenic artery aneurysm has a female predominance, can be diagnosed at any age, but is more commonly seen in the fifth and sixth decades with a mean age of presentation of 52 years [3]. In our case series we had male predominance as most of them were giant (>5 cm). Giant SAAs are 1.78 times more frequent in males [5].

Dr. Chaer commented in the society for vascular surgery announcement, ‘Nearly one-fourth of visceral artery aneurysms reported have presented with rupture and the mortality rate of these diagnosed ruptures is at least 10%, probably higher. The mortality following ruptured celiac artery aneurysms and ruptured splenic artery aneurysms in pregnant women approaches 100%.’ [6].

**Table 2:** Recommendations for treatment of SAA

Recommendation	Strength of recommendation	Quality of evidence
In patients with ruptured SAA discovered at laparotomy, we suggest treatment with ligation with or without splenectomy, depending on the aneurysm location	2 (weak)	B (moderate)
In patients with ruptured SAA diagnosed on preoperative imaging studies, we suggest treatment with open surgical or appropriate endovascular techniques based on the patient’s anatomy and underlying clinical condition	2 (weak)	B (moderate)
We suggest elective treatment of SAA using an endovascular approach if it is anatomically feasible. However, elective treatment may appropriately involve open surgical, endovascular or laparoscopic methods of intervention, depending on the patient’s anatomy and underlying clinical condition	2 (weak)	B (moderate)
In treatment of SAA, we suggest that the splenic artery does not routinely require preservation or revascularization	2 (Weak)	C (low)
In treatment of distal SAA adjacent to the hilum of the spleen, we suggest open surgical techniques including possible splenectomy as opposed to endovascular methods, given concern for the possibility of end organ ischemia, including splenic infarction and pancreatitis	2 (Weak)	C (low)
In pregnant women with SAA, treatment decisions should be individualized regardless of size, and the potential morbidity to both them other and fetus should be considered	Ungraded best practice statement	

The most frequent management options for SAAs are medical treatment, close follow-up, open surgery, endovascular treatment and laparoscopic surgery [5]. Management of SAAs depends on various factors including age, gender, aneurysm dimension, origin and the severity of the clinical findings and their complications [6–7]. Since the spleen has abundant collateral circulation, simple ligation of the main trunk of the splenic artery does not cause major complications so in four of our patients with proximal middle part involvement, we resected the aneurysm without splenectomy. As we carried out in our sixth patient, aneurysmectomy with end-to-end anastomosis can be performed safely. This method preserves the spleen, an important element of the immune system.

If the SPAA is located in one-third of the peripheral region of the artery (first patient) or in the vessel that branches of the splenic hilum, then vascular reconstruction is difficult, in which case, splenectomy is usually performed.

One of the major benefits of endovascular treatment (EVT) is that it is minimally invasive. It can be performed using local anesthesia and is followed by early postoperative recovery and consequently, a shorter hospital stay. Thus, EVT is effective for high-risk patients with multiple comorbidities and those with a history of abdominal surgery, for whom intraperitoneal adhesion is a concern. The primary limitations of EVT are the lack of availability of facilities with adequate resources for emergency treatment, the risk of access-related injury and end-organ embolization, contrast toxicity and the need for prolonged imaging surveillance [8].

There are two embolization techniques: endovascular ligation that requires the positioning of the coils on either side of the aneurysm (‘sandwich technique’) to attain complete occlusion. For patients with large or multiple SAAs, total embolization of the splenic artery must be performed to produce complete occlusion of the SAAs [9]. In cases 4 and 5 we started EVT but as they were giant aneurysms we continued by laparotomy (the hybrid surgery).

Chaer *et al*. [6] published the latest guideline in society for vascular surgery and declared recommendations to manage SAA (Table 2). They also suggested screening of patients with SAAs for other intra-abdominal, intrathoracic, intracranial and peripheral artery aneurysms [6].

## CONCLUSION

Endovascular procedures considered as a first choice of treatment for splenic artery aneurysm. Open surgery is reserved mostly for the treatment of complications or if the endovascular techniques fail, lack of availability of endovascular procedures or allergy to contrast medium. Surgical procedure**s** for splenic artery aneurysm may involve splenic artery ligation, aneurysmectomy or splenectomy. Improvements in endovascular therapies have also allowed an enhanced ability for treatment of anatomically complex lesions with a large variety of individualized and precise catheter-based therapies**.**

## CONFLICT OF INTEREST STATEMENT

We have no conflicts of interest to declare.

## FUNDING

None.
